# Beclin 1 Expression in Ovarian Tissues and Its Effects on Ovarian Cancer Prognosis

**DOI:** 10.3390/ijms15045292

**Published:** 2014-03-26

**Authors:** Mingbo Cai, Zhenhua Hu, Juanjuan Liu, Jian Gao, Chuan Liu, Dawo Liu, Mingzi Tan, Danye Zhang, Bei Lin

**Affiliations:** Department of Obstetrics and Gynecology, Shengjing Hospital Affiliated to China Medical University, No. 36 Sanhao Street, Heping District, Shenyang 110004, Liaoning, China; E-Mails: caimingbo@outlook.com (M.C.); huzh5062012@163.com (Z.H.); liujj@sj-hospital.org (J.L.); gaoxiaojian@sina.com (J.G.); liuchuan@sina.com (C.L.); cyldw2007@163.com (D.L.); kitefly1102@126.com (M.T.); zhangdanye624@163.com (D.Z.)

**Keywords:** autophagy, Beclin 1, ovarian cancer, prognosis, overall survival

## Abstract

Beclin 1 is an autophagy-associated protein involved in apoptosis and drug resistance, as well as various malignancies. We investigated the expression of Beclin 1 protein in ovarian epithelial tissues and correlated it with the prognosis of ovarian cancer. Beclin 1 protein expression was determined using immunohistochemistry in 148 patients with ovarian epithelial cancer, 26 with ovarian borderline tumor, 25 with benign ovarian tumor, and 30 with normal ovarian tissue. The relationships between Beclin 1 protein expression and ovarian cancer pathological characteristics were analyzed. The risk factors for ovarian cancer prognosis were analyzed using Cox’s regression model. A survival curve was plotted from the follow-up data of 93 patients with ovarian cancer to analyze the effects of Beclin 1 expression on the prognosis of ovarian cancer. The positive rates of Beclin 1 were significantly higher in ovarian epithelial cancer (148) and borderline tumor (26) than in benign ovarian tumor (25) or normal ovarian tissue (30) (all *p* < 0.001). The surgical stage and Beclin 1 expression were both independent risk factors for ovarian cancer prognosis (both *p* < 0.05). Patients with high Beclin 1 levels showed better survival than those with low Beclin 1 levels (*p =* 0.009). Beclin 1 protein is upregulated in ovarian epithelial cancer and is a prognostic factor of ovarian cancer.

## 1. Introduction

The mammalian *BECN1* gene is homologous to the yeast *ATG6* and is located at chromosome 17q21 in humans. *BECN1* was the first-characterized mammalian gene involved in the initiation of autophagy [1]. Liang *et al.* found that overexpression of *BECN1* gene in the MCF-7 breast cancer cell line reversed malignant phenotype and inhibited cell proliferation by enhancing autophagy. They also showed that MCF-7 cells overexpressing Beclin 1 had a reduced capacity to form tumors in nude mice [2]. Yue reported that *BECN1*^+/−^ nude mice showed reduced autophagic activity and an increased incidence of spontaneous tumors, including lymphoma, hepatocellular carcinoma and lung adenocarcinoma [3]. These results indicate that suppressive autophagy contributes to tumorigenesis. *BECN1* is a tumor suppressor gene and is involved in autophagy and tumor cell apoptosis. Furuya found that Beclin 1 can promote the apoptosis induced by the chemotherapeutic drug cis-diamminedichloroplatinum (CDDP) in the MKN28 human gastric cancer cell line by enhancing the caspase 9 activity [4]. Sun reported that the CaSki cervical cancer cell line overexpressing Beclin 1 displayed reduced proliferation and increased apoptotic sensitivity to paclitaxel [5].

Beclin 1 is differentially expressed in various malignancies, suggesting that it plays different roles in these tumors. For example, Beclin 1 expression is upregulated in gastric cancer, colorectal cancer, intrahepatic cholangiocellular carcinoma, and extranodal natural killer T-cell lymphoma [6–8], but is downregulated in high-grade gliomas, nasopharyngeal carcinoma, and esophageal squamous cell carcinoma [9–11]. However, the relationship between the level of Beclin 1 expression and cancer prognosis is not yet conclusive. The overexpression of Beclin 1 is associated with a good prognosis in stage IIIB colon cancer [12], but with a poor prognosis in nasopharyngeal carcinoma [10]. Many recent studies have investigated the levels of Beclin 1 expression in malignant tumors and the relationship between these level and the prognoses of these tumors. However, whether the level is high or low in ovarian cancer is controversial, and the relationship between Beclin 1 expression and its prognosis is still unclear. In this study, we investigated the relationship between the level of Beclin 1 expression and prognosis in ovarian cancer.

## 2. Results

### 2.1. Beclin 1 Expression in Ovarian Tissues

**Figure 1.**
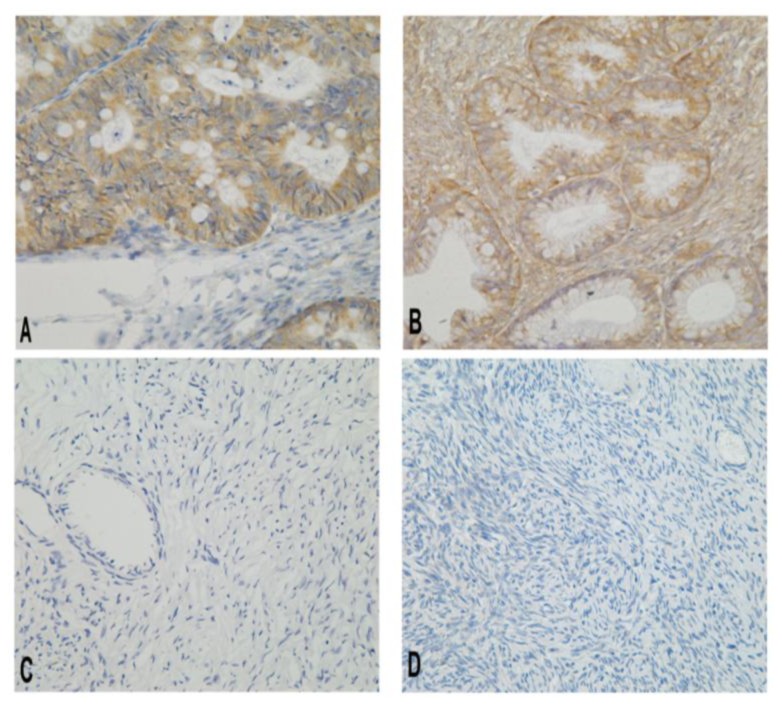
Immunohistochemical micrographs of Beclin 1 protein in different ovarian tissues (400×). The level of Beclin 1 was lower in normal ovarian tissue and benign ovarian epithelial tissue than in ovarian epithelial cancer or borderline ovarian tumor. (**A**) Malignant; (**B**) borderline; (**C**) benign; and (**D**) normal ovarian tissue.

Beclin 1 immunoreactivity was predominantly found in the cytoplasm, but also in the nuclei and on membranes (Figure 1A,B). The positive expression rate of Beclin 1 was 80.41% in ovarian epithelial cancers, 73.08% in borderline tumors, 23.08% in benign tumors, and 20.00% in normal ovarian tissues (Table 1). The positive rates of Beclin 1 were significantly higher in ovarian epithelial cancer and borderline tumor than in benign ovarian tumor or normal ovarian tissue (all *p* < 0.001; Table 1).

### 2.2. The Expression of Beclin 1 Protein in Fresh Ovarian Tissues

**Figure 2.**
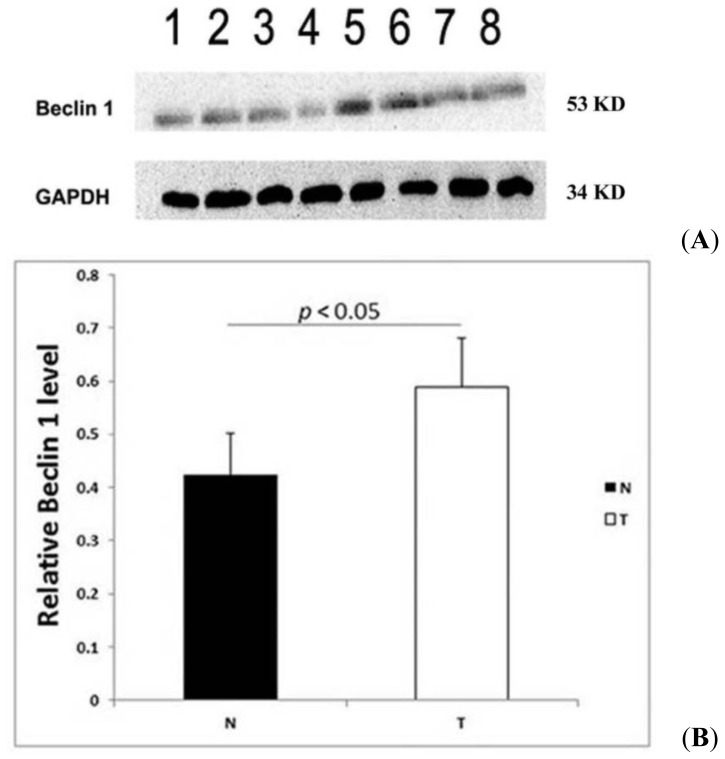
The expression of Beclin 1 protein in fresh ovarian tissues. (**A**) Rows **1**–**4**: normal ovarian tissues; and rows **5**–**8**: ovarian carcinoma tissues. Beclin 1 protein expression was elevated in ovarian carcinoma tissues compared with normal ovarian tissues on immunoblotting; and (**B**) quantitative data for normal ovarian tissues and ovarian carcinoma tissues. N indicates normal ovarian tissues (*n* = 10); and T indicates ovarian carcinoma tissues (*n* = 20). The expression of Beclin 1 protein in ovarian carcinoma tissues was elevated compared with that in normal ovarian tissues (*p* < 0.05).

We also examined the ovarian carcinoma tissues of 20 patients, including 15 serous adenocarcinomas and five poorly differentiated adenocarcinomas, and 10 samples of normal ovarian tissues (excised during the surgical removal of cervical cancers). We used immunoblotting to detect the expression of Beclin 1 at the protein level and found that the levels of Beclin 1 were higher in the ovarian carcinoma than in the normal ovarian tissues (Figure 2A,B).

### 2.3. Relationship between Beclin 1 Levels and Ovarian Cancer Pathology

In total, 148 cases of ovarian cancer were divided into a Beclin 1 high-expression group (++/+++) and a Beclin 1 low-expression group (−/+). The rate of high Beclin 1 expression differed significantly between patients with well-moderately differentiated ovarian cancers (69.1%) and those with poorly differentiated cancers (51.9%; *p* < 0.05). However, the Beclin 1 levels were not associated with pathological type, surgical stage, or lymph-node metastasis (all *p* > 0.05; Table 2).

### 2.4. Risk Factors for Ovarian Cancer Prognosis

Cox’s regression analysis was performed using surgical stage, differentiation, pathological type, lymph-node metastasis, residual lesion size, and Beclin 1 expression as the dependent variables, and survival time as the independent variable. The results showed that surgical stage and Beclin 1 expression were independent risk factors for ovarian cancer prognosis (Table 3).

### 2.5. Survival Analysis

**Figure 3.**
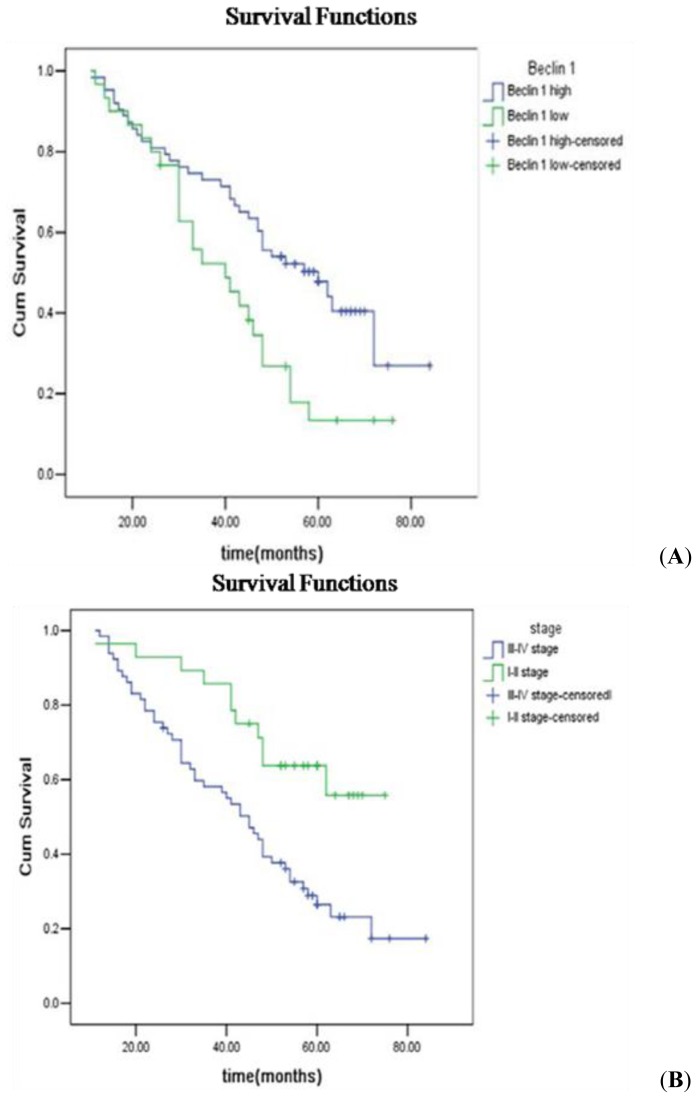
The association between overall survival, Beclin 1 expression, and surgical stage in 93 patients with ovarian cancer. (**A**) Patients with high Beclin 1 expression showed significantly longer overall survival than those with low Beclin 1 expression (*p* = 0.009); and (**B**) Patients with stage I–II disease showed significantly longer overall survival than those with stage III–IV disease (*p* = 0.003).

As of December 2013, the follow-up times ranged from 54 months to 103 months, and 24 deaths occurred in the Beclin 1 low-expression group (30 patients) and 35 deaths in the high-expression group (63 patients). A Kaplan–Meier analysis showed that the survival rate was significantly higher in the Beclin 1 high-expression group than in the low-expression group (*p <* 0.05; Figure 3A). The survival rate in patients with stage III–IV ovarian cancer (48 deaths) was significantly lower than that in patients with stage I–II disease (11 deaths; *p <* 0.05; Figure 3B).

## 3. Discussion

Autophagy is the lysosomal degradation of excessive proteins and subcellular structures, and is also known as type II programmed cell death. It is well established that autophagy is involved in tumor formation and development [13]. Beclin 1 mediates the anchoring of other autophagic proteins in the preautophagosomes and regulates autophagy, apoptosis, and cell differentiation [14]. The expression of Beclin 1 and its roles in malignant tumors have drawn great interest. In this study, using large samples of tissues from the northeast of China, we found that the positive expression rate of Beclin 1 in ovarian epithelial cancers (80.41%) was significantly higher than that in benign ovarian tumors (23.08%) or normal ovarian tissues (20.00%) (all *p* < 0.001). We used immunoblotting to compare the overexpression of Beclin 1 in ovarian carcinomas at the protein level with that in normal ovarian tissues. Duan *et al.* [15,16] reported that Beclin 1 expression was low in ovarian carcinomas, which is contrary to our result. We consider that this discrepancy can be attributed to the following factors: (1) *BECN 1* mRNA has three variant transcripts, which may be translated into different subtypes of the protein. We used a different anti-Beclin 1 antibody from that used by Duan and Shen, which may have produced different results; (2) the sources of the patient samples differed. Our patients were mainly from the northeast of China, whereas the patients of Duan *et al*. were mainly from the south of China. This difference may have directly affected the results of the two analyses. It has been shown that Beclin 1 is overexpressed in gastric cancer and colorectal cancer [6], which is consistent with our results. However, Beclin 1 expression is downregulated in other malignancies, such as breast cancer and esophageal squamous cell carcinoma [2,11], suggesting different roles for Beclin 1 in different cancers. Futreal *et al.* performed genetic assays in 21 patients with primary ovarian cancer (Caucasians and African-Americans) and found a single-allele deletion of BECN1 in 12 cases [17], but there have as yet been no reports of the occurrence of this allele detection in the Asian populations. In the present study, Beclin 1 expression correlated positively with ovarian cancer differentiation in Asian patients (*p* < 0.05), suggesting that Beclin 1 is a protective factor in ovarian cancer.

Whether Beclin 1 expression is associated with the prognosis of ovarian cancer is not yet clear. In 2013, Zhao *et al.* [18] undertook a multivariant survival analysis of the prognosis of ovarian carcinoma, and concluded that Beclin 1 is not an independent risk factor for ovarian carcinoma prognosis. However, the shortest follow-up period in their study was only one month. The shortest follow-up period in our study was 54 months and the median follow-up period was 69 months, so our follow-up period was more normative than that of Zhao *et al.* In this study, we explored the relationship between the expression of Beclin 1 and the prognosis of ovarian carcinoma again and found that Beclin 1 expression and surgical stage are independent risk factors for this prognosis. A survival analysis showed that patients with high Beclin 1 expression survived significantly longer than those with low Beclin 1 expression, suggesting that the *BECN1* gene might be a tumor suppressor gene and that Beclin 1 levels are associated with the prognosis of ovarian cancer. Similar protective effects of Beclin 1 have also been reported for gastric cancer, intrahepatic cholangiocellular carcinoma, extranodal natural killer T-cell lymphoma, and stage IIIB colon cancer [6–8,12].

There has been increasing research into the effects of Beclin 1 on cancer progression in recent years, but no definite conclusions have been established. Kenji *et al.* showed that the stable transfection of the CaSki cervical cancer cell line or the HT29 colon cancer cell line with the *BECN1* gene significantly suppressed cell proliferation and increased the apoptotic sensitivity of CaSki cells to paclitaxel [5,19]. In the HTC116 colon cancer line, the c-jun *N*-terminal Kinase (JNK) signaling pathway upregulates Beclin 1 expression, enhances the phosphorylation of p53 and Bcl-2, and finally promotes autophagic death [20]. Beclin 1 promoted the apoptosis induced by the chemotherapeutic drug CDDP in the MKN28 human gastric cancer cell line by enhancing caspase 9 activity [4]. Yin *et al.* showed that the upregulated expression of Beclin 1 significantly inhibited the proliferation of leukemia cells by promoting autophagy [21]. Reduced Beclin 1 expression in hepatocellular cancer is often accompanied by the increased expression of antiapoptotic Bcl-XL, increasing the survival of hepatocellular cancer cells. The interaction between Bcl-XL and Beclin 1 can inhibit autophagy and thus promote the progression of malignant tumors [22]. Our study shows that high Beclin 1 levels are associated with a lower risk of death in patients with ovarian cancer. The possible mechanisms include: (1) Beclin 1 induces autophagy in ovarian cancer cells lacking apoptotic ability; (2) Beclin 1 stabilizes the mitochondrial structure and reduces the frequency of additional gene mutations [23]; and (3) Beclin 1 overexpression arrests the cell cycle, inhibits cell proliferation, and promotes autophagy and apoptosis.

In summary, Beclin 1 protein is overexpressed in ovarian cancer and may play an important role in the development of this malignancy. Further study is required to investigate the possibility of targeting Beclin 1 in the treatment of ovarian cancer.

## 4. Materials and Methods

### 4.1. Patients and Follow-up

Written informed consent was not given by the participants for their clinical records and tissue samples to be used in this study. However, the patient information was anonymized and de-identified before its analysis. The analysis of patient tissues was approved by the Institutional Review Board of Shengjing Hospital of China Medical University (the ethics approval code was “2013PS158K”).

Ovarian tumors (malignant, borderline, and benign) and normal ovarian tissues from patients treated between 2004 and 2009 at Shengjing Hospital, affiliated with the China Medical University, were collected retrospectively. All tissue sections were examined by specialists to make a final diagnosis. Histopathological diagnoses were made using the World Health Organization criteria. The classification of cancer stage and grade was according to the International Federation of Gynecology and Obstetrics (FIGO, 2009). There were 148 cases of primary malignant ovarian tumors, 26 borderline ovarian tumors, 25 benign ovarian tumors, and 30 normal ovarian tissues (excised during the surgical removal of cervical cancers). The clinical and pathological information about the patients was collected from their clinical records, and included their age, surgical stage, lymph-node metastasis, pathological tumor grade and subtype, and residual tumor size.

The age range (median) was 16–76 years (52.7 years) in the malignant ovarian tumor group; 22–77 years (39.3 years) in the borderline ovarian tumor group; 15–81 years (43 years) in the benign ovarian tumor group; and 37–62 years (45.5 years) in the normal ovarian tissue group. There were no statistically significant differences in the ages of these groups (*p* > 0.05). The specific histological types and pathological grades are shown in Tables 1 and 2.

We collected information on the clinical chemotherapeutic treatments received and the follow-up of 93 patients from a total 148 patients with malignant ovarian cancer. These 93 patients underwent treatment for ovarian cancer that included surgical debulking followed by 6–8 postoperative cycles of conventional chemotherapy consisting of paclitaxel (Taxol^®^, Harbin pharmaceutical group, Harbin, China) plus carboplatin and were followed-up for a minimum of three years after the completion of chemotherapy. We defined the overall survival of the patients as extending from the date of surgery to the date of death or the last follow-up. After the operation, patients were observed at 6-month intervals. To determine the factors influencing survival after surgery and standard chemotherapy, conventional variables together with Beclin 1 expression were tested in 93 ovarian carcinoma patients.

### 4.2. Immunohistochemistry

Paraffin-embedded histological sections of each group of ovarian tissues were cut to 5 μm. Immunohistochemistry was used to analyze the Beclin 1 expression levels. Rabbit monoclonal anti-Beclin 1 antibody (ab51031; diluted 1:180) was bought from Abcam Company (Cambridge, UK). The staining procedure was performed according to the manual of an ultrasensitive streptavidin–peroxidase kit (MaiXin.Bio, Kit 9701, Fuzhou, China) and Harris’s hematoxylin (Sairuida.Bio, Tianjin, China) was used to stain the cell nuclei. Tissues treated with phosphate-buffered saline (PBS) instead of the primary antibody were used as the negative control. A colon cancer tissue sample was used as the positive control. Buff-colored granules in the cell cytoplasm and nucleus were considered a positive result. The tissues were rated according to their chromatic intensity: no pigmentation = 0, light yellow = 1, buff = 2, and brown = 3. We chose five high-power fields in serial sections from each slice, scored them, and estimated the mean percentage of chromatic cells: <5% chromatic cells = 0; 5%–25% chromatic cells = 1; 26%–50% chromatic cells = 2; 51%–75% chromatic cells = 3; and >75% chromatic cells = 4. We multiplied these two numbers (intensity score × percentage chromatic cells): 0–2 was considered (−); 3–4 (+); 5–8 (++), and 9–12 (+++). The total 148 cases of ovarian cancer were divided into the Beclin 1 high-expression group (++/+++) and the Beclin 1 low-expression group (−/+). Two observers read the sections to control for error.

### 4.3. Immunoblotting

The soluble proteins were isolated from the tissues for western blotting. The protein concentrations was measured by Bicinchoninic acid (23228, Thermo Fisher Scientific, Waltham, MA, USA). Equal amounts of protein from each sample were separated by electrophoresis on an SDS-10% polyacrylamide gel, transferred to polyvinylidene difluoride membrane, and blocked with 5% nonfat dry milk in 1× TBS plus 0.1% Tween 20 at room temperature for 2 h. The membranes were incubated overnight at 4 °C with the primary antibody in 1% albumin bovine in 1× TBS plus 0.1% Tween 20. The primary anti-Beclin 1 monoclonal antibody (diluted 1:2000) was purchased from Abcam Company (ab51031) and the anti-GAPDH (glyceraldehyde-3-phosphate dehydrogenase) monoclonal antibody (diluted 1:2000) was purchased from Boshide Biotech Company (BM1985, Wuhan, China). The membranes were washed and incubated again for 2 h at room temperature with horseradish-peroxidase-conjugated anti-rabbit or anti-mouse secondary antibody. The proteins were visualized with ECL reagent (ECL Prime Western Blotting Detection Reagent, Amersham, Pittsburgh, PA, USA). The experiments were repeated three times.

### 4.4. Statistical Analyses

Differences in proportions were evaluated using the *χ*^2^ test. The *χ*^2^ test or Fisher’s exact test was used to analyse the relationship between the Beclin 1 expression and clinicopathological variables, whichever was appropriated. Survival curves were generated using the Kaplan–Meier method and compared by the log-rank test. Cox’s proportional hazard regression model was used for multivariate survival analysis of prognostic factors. All statistical analyses were performed using SPSS V17.0 software (SPSS Inc., Chicago, IL, USA). A two-tailed *p*-value test was used in all analyses; *p*-values less than 0.05 were considered statistically significant.

## 5. Conclusions

Beclin 1 protein is upregulated in ovarian epithelial cancer and is a prognostic factor for this cancer.

## Figures and Tables

**Table 1.** Expression of Beclin 1 in different ovarian tissues.

Groups	Cases	Beclin 1				Positive cases	Positive rates (%)

		−	+	++	+++		
Malignant	148	29	26	56	37	119	80.41 *
Borderline	26	7	10	6	3	19	73.08 *
Benign	25	19	5	1	0	6	23.08
Normal	30	24	5	1	0	6	20.00

* Compared with the benign group or normal group. *p* < 0.001.

**Table 2.** Relationships between the expression of Beclin 1 protein and clinicopathological characteristics of 148 patients with malignant ovarian cancer.

Characteristics	No.	Beclin 1 expression		
	
	Cases (%)	Low	High	*p*-value
**Pathologic type**				*p* > 0.05

Serous	93 (62.8%)	35 (37.6%)	58 (62.4%)	
Mucinous	28 (18.9%)	9 (32.1%)	19 (67.9%)	
Endometrioid	9 (6.1%)	5 (55.6%)	4 (44.4%)	
Clear cell carcinomal	7 (4.7%)	2 (28.6%)	5 (71.4%)	
Poorly differentiated adenocarcinoma	11 (7.5%)	3 (27.3%)	8 (72.7%)	

**Surgical stage**				*p* > 0.05

I–II	65 (43.9%)	24 (36.9%)	41 (63.1%)	
III–IV	83 (56.1%)	31 (37.3%)	52 (62.7%)	

**Differentiation**				*p* < 0.05

Well-moderate	94 (63.5%)	29 (30.9%)	65 (69.1%)	
Poor	54 (36.5%)	26 (48.1%)	28 (51.9%)	

**Lymph node metastasis** *				*p* > 0.05

No	90 (78.9%)	35 (38.9%)	55 (61.1%)	
Yes	24 (21.1%)	6 (25.0%)	18 (75.0%)	

* Thirty four cases of ovarian carcinoma patients without lymph node resection.

**Table 3.** Multivariate analysis of the prognosis of patients with ovarian carcinoma.

Variables	*p*-value	Hazard radio (95% CI)
Surgical stage (I + II *vs.* III + IV)	0.046	2.133 (1.014–4.489)
Beclin 1 (low *vs.* high)	0.037	0.557 (0.322–0.965)
